# An Optical Image Encryption Method Using Hopfield Neural Network

**DOI:** 10.3390/e24040521

**Published:** 2022-04-07

**Authors:** Xitong Xu, Shengbo Chen

**Affiliations:** College of Geo-Exploration Science and Technology, Jilin University, Changchun 130026, China; xitong19@mails.jlu.edu.cn

**Keywords:** optical image encryption, wavelet packet transform, double random phase encoding, 4*f* system, Fresnel domain, Hopfield neural network, single neuronal dynamic system

## Abstract

In this paper, aiming to solve the problem of vital information security as well as neural network application in optical encryption system, we propose an optical image encryption method by using the Hopfield neural network. The algorithm uses a fuzzy single neuronal dynamic system and a chaotic Hopfield neural network for chaotic sequence generation and then obtains chaotic random phase masks. Initially, the original images are decomposed into sub-signals through wavelet packet transform, and the sub-signals are divided into two layers by adaptive classification after scrambling. The double random-phase encoding in 4*f* system and Fresnel domain is implemented on two layers, respectively. The sub-signals are performed with different conversions according to their standard deviation to assure that the local information’s security is guaranteed. Meanwhile, the parameters such as wavelength and diffraction distance are considered as additional keys, which can enhance the overall security. Then, inverse wavelet packet transform is applied to reconstruct the image, and a second scrambling is implemented. In order to handle and manage the parameters used in the scheme, the public key cryptosystem is applied. Finally, experiments and security analysis are presented to demonstrate the feasibility and robustness of the proposed scheme.

## 1. Introduction

In the development of digital technology and computer industry, the requirements for information confidentiality has attracted increasing attention. In order to provide protection to multimedia applications, many algorithms have been developed during the past several decades. The application of optical methods in information security has become a hot trend due to its inherent capabilities of parallel processing [1,2,3,4,5]. The classic double random-phase encoding was proposed based on an optical Fourier transform system. Subsequently, a large number of approaches have been developed to searching for other types of optical encryption methods in different domains, such as fractional Fourier domain [6,7,8], Fresnel domain [9,10] and gyrator domain [11]. However, due to inherent symmetry and linearity, the security of such cryptosystems is greatly affected [12,13]. During the process of optical image encryption, it is necessary not only to conceal the information of whole image, but also to selectively encrypt the important information, so as to improve local security. In addition, considering the characteristics of chaotic systems (i.e., sensitivity to initial values, deterministic dynamics, nonlinear transformation and pseudo-randomness) [14,15], applying chaotic systems to optical image encryption may have a positive impact on the overall security.

Hopfield neural network, proposed for the first time by Hopfield in 1982 [16], is a typical dynamic neural network which has been applied in information processing and engineering, such as associative memory [17] and optimization problems [18]. It is worth noting that the Hopfield neural network plays a crucial role in neuro-computing due to its similarity to brain dynamics [19], and the complex behaviors and properties of the Hopfield neural network have been investigated [20,21,22,23]. As more and more research is done to combine chaos theory and information security, neural networks have become a vital method to be considered in image encryption. Particularly, a Hopfield neural network with chaos can greatly improve the space–time complexity of an encryption scheme through outstanding nonlinear and associative memory effects [24,25,26,27]. However, few studies have integrated the Hopfield neural network and optical methods to enhance the security of information.

In recent years, chaotic systems have been combined with different cryptosystems and technologies for image encryption, such as compression sensing [28,29] and DNA [30,31]. The combination has been proved to effectively improve the key space and the stability of encryption algorithms [32,33], and plenty of simple chaotic systems (e.g., logistic map and sine map) have been applied due to uncomplicated structure [34,35,36]. Nevertheless, the security of optical image encryption cannot be improved by using simple chaotic systems on account of their structure and insufficient parameters. Moreover, the sensitivity to computer precision may cause the systems to degenerate into non-chaotic systems immediately [37]. The single neuronal dynamic system was derived from the Hopfield neural network by Xu et al. in 2021 [38]. The system has sufficient parameters and complex chaotic dynamical behavior, whereas chaos cannot always be maintained in the interval of some parameters. Fuzzy numbers have a large field of study with applications in dynamical systems, which promote the systems to present many chaos-related phenomena [39,40,41]. The single neuronal dynamic system also has the applicability to combine with fuzzy numbers to further enhance the stability and chaotic phenomena.

In this paper, a chaotic Hopfield neural network and fuzzy single neuronal dynamic system are combined with a hybrid optical method to construct a new encryption method. In this scheme, the input image is decomposed into sub-signals through wavelet packet transform, and the sub-signals are divided into two layers by adaptive classification after scrambling. The chaotic random-phase masks are constructed by chaotic sequences. The first layer of sub-signals is encrypted by double random phase encoding (DRPE) in 4*f* system, and the second layer is encrypted by double random phase encoding in Fresnel domain. After inverse wavelet packet transform, the obtained image is secondarily scrambled. In addition, the keys used in the process of encryption are integrated and hidden by RSA cryptosystem. Finally, simulation experiments demonstrate the feasibility and security of the proposed method.

## 2. Related Chaotic System and Public Key Cryptosystem

### 2.1. Fuzzy Single Neuronal Dynamical System

The single neuronal dynamical system in Hopfield neural network was proposed by Xu et al. in 2021 [38]. The mathematical model of the system is described as follows:

(1) $$\left\{\begin{array}{c} v_{i}\left(t\right)=\frac{1}{1+exp\left(-\gamma u_{i}\left(t\right)\right)}\\ v_{i}^{\prime }\left(t\right)=v_{i}\left(t\right)\times 2^{n}-floor\left(v_{i}\left(t\right)\times 2^{n}\right)\\ u_{i}\left(t+1\right)=ku_{i}\left(t\right)+zv_{i}^{\prime }\left(t\right)+h \end{array}\right.$$

where γ, k, z, h and n are system parameters. The robustness and sensitivity of single neuronal dynamical system has been verified in [38]. However, the performance of single neuronal dynamical system can be further improved by using fuzzy numbers.

In this work, we consider the triangular form of fuzzy number as Equation (2), and the full presentation of fuzzy number can be found in [42,43,44].

(2) $$f_{s}\left(x\right)=\left\{\begin{array}{c} \frac{x}{s}\;0\leq x\leq z\\ \frac{1-x}{1-s}\;z\leq x\leq 1 \end{array}\right.$$

where s is the peak of the triangular fuzzy number.

We propose partitioning single neuronal dynamical system by combining it with the triangular form of fuzzy number to generate fuzzy single neuronal dynamical system fuzzy single neuronal dynamic system, as shown in Equation (3).

(3) $$\left\{\begin{array}{c} v_{i}\left(t\right)=f_{s}(\frac{1}{1+\exp\left(-\gamma u_{i}\left(t\right)\right)})\\ v_{i}^{\prime }\left(t\right)=v_{i}\left(t\right)\times 2^{n}-floor\left(v_{i}\left(t\right)\times 2^{n}\right)\\ u_{i}\left(t+1\right)=ku_{i}\left(t\right)+zv_{i}^{\prime }\left(t\right)+h \end{array}\right.$$

**Figure 1:**
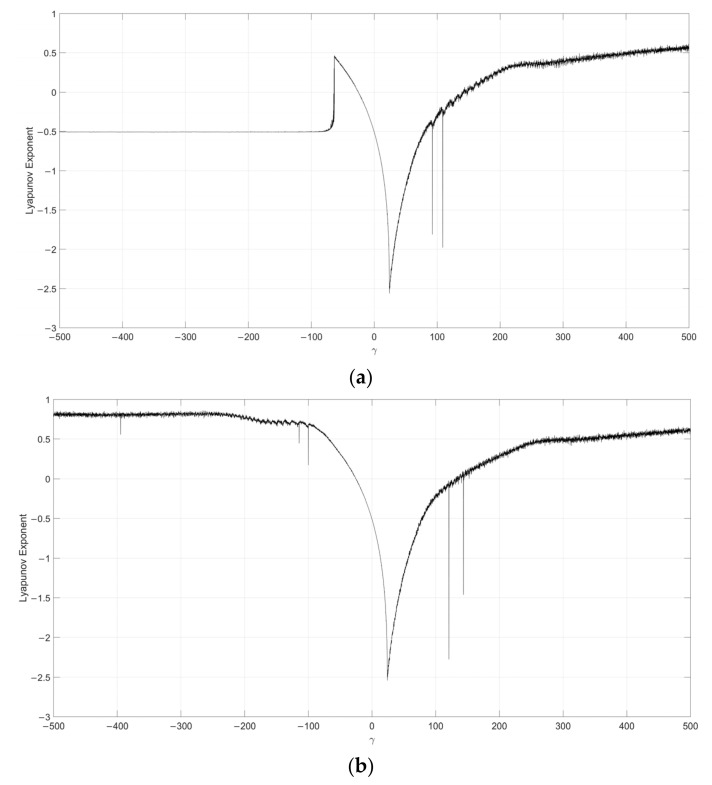
Lyapunov exponent diagram of parameter y when s=0.1, k=0.5, z=−0.1, h=0.01 and n=14 for (**a**) single neuronal dynamic system and (**b**) fuzzy single neuronal dynamic system.

**Figure 2:**
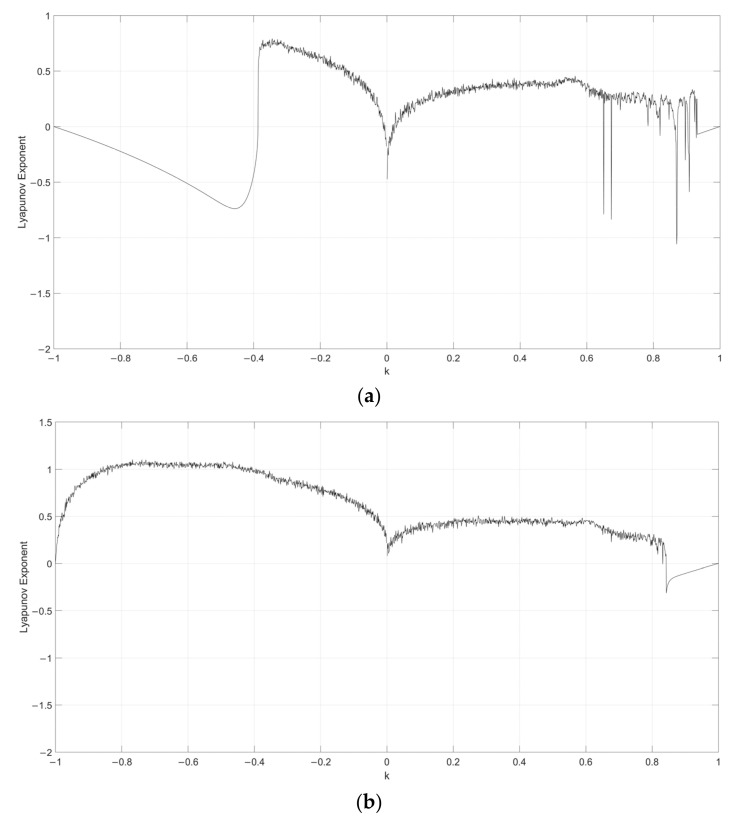
Lyapunov exponent diagram of parameter k when s=0.1, y=250, z=−0.1, h=0.01 and n=14 for (**a**) single neuronal dynamic system and (**b**) fuzzy single neuronal dynamic system.

**Figure 3:**
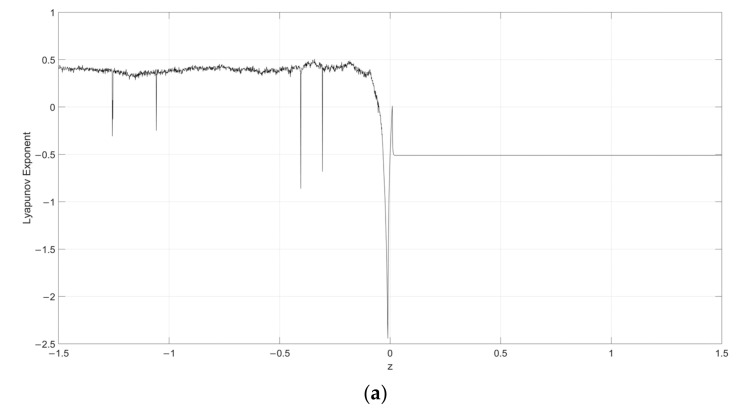

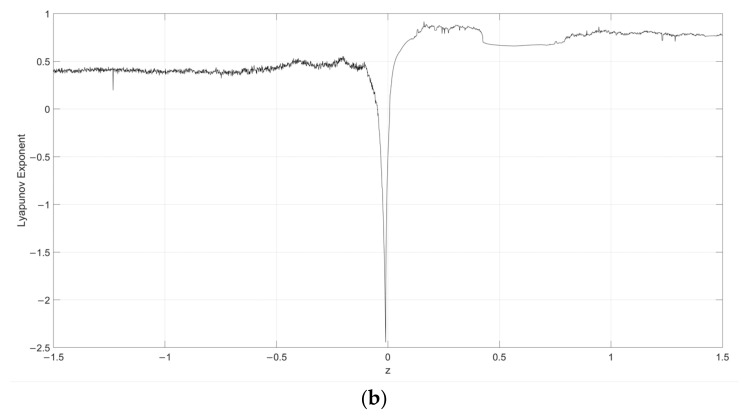
Lyapunov exponent diagram of parameter z when s=0.1, y=250, k=0.5, h=0.01 and n=14 for (**a**) single neuronal dynamic system and (**b**) fuzzy single neuronal dynamic system.

**Figure 4:**
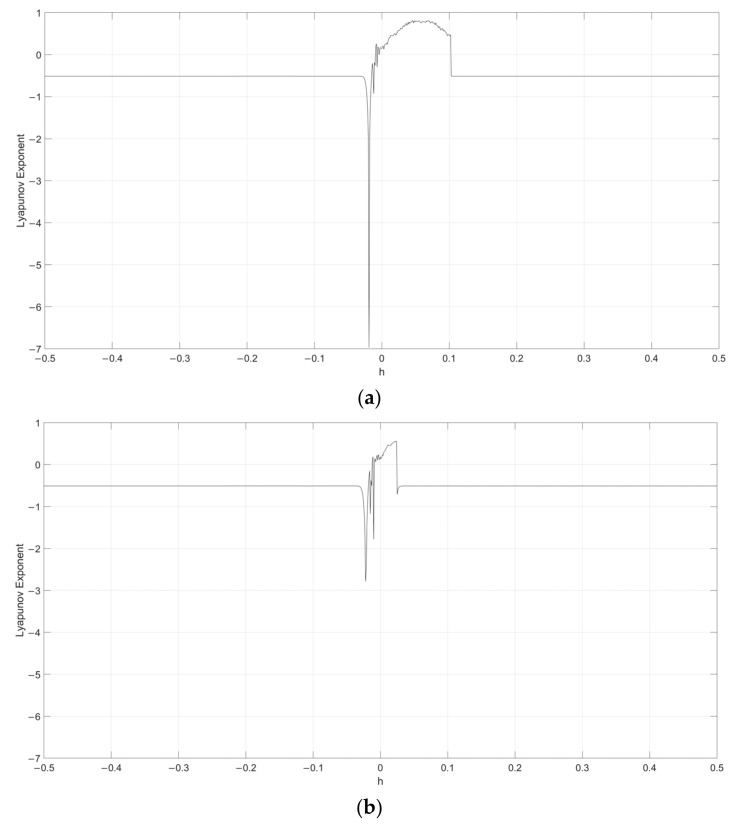
Lyapunov exponent diagram of parameter h when s=0.1, y=250, k=0.5, z=−0.1 and n=14 for (**a**) single neuronal dynamic system and (**b**) fuzzy single neuronal dynamic system.

**Figure 5:**
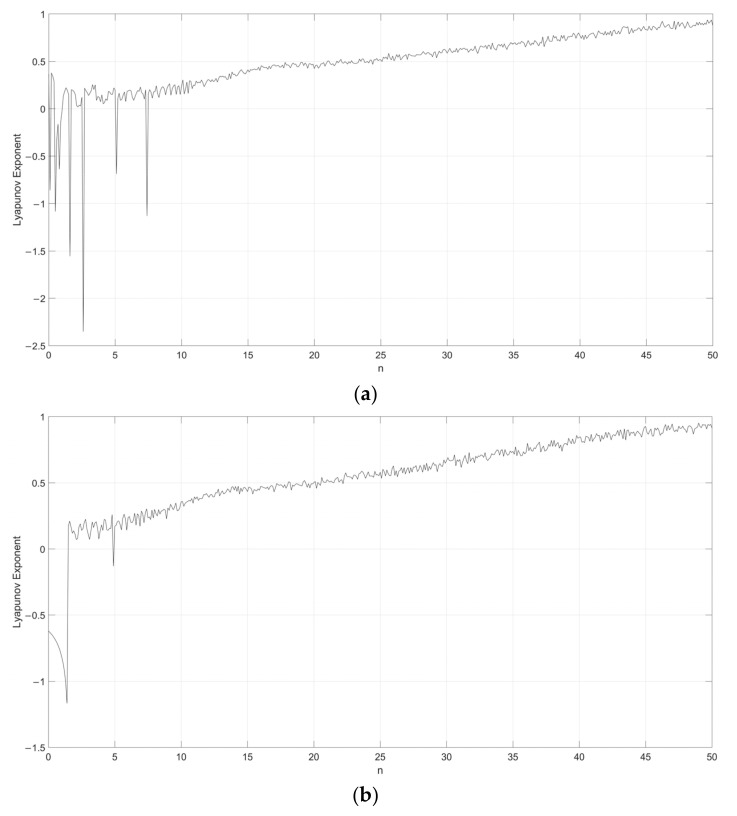
Lyapunov exponent diagram of parameter n when s=0.1, y=250, k=0.5, z=−0.1 and h=0.01 for (**a**) single neuronal dynamic system and (**b**) fuzzy single neuronal dynamic system.

Considering s=0.1, the Lyapunov exponent evolution comparison between the single neuronal dynamical system and the fuzzy single neuronal dynamic system is performed, as shown in Figure 1, Figure 2, Figure 3, Figure 4 and Figure 5. It can be seen that the stability of the fuzzy single neuronal dynamic system and the interval in the chaotic state are significantly increased. For parameter γ, Figure 1 shows the instances of entering the chaotic state at γ>132.8 and γ<−25.3, which indicates there are larger chaos intervals on the both sides of the zero point. A similar phenomenon is also observed in parameters k and z. For parameter n, as shown in Figure 4, its Lyapunov exponent fluctuates more smoothly, which is similar to parameters *k* and *z*.

### 2.2. Hopfield Chaotic Neural Network

This paper considers a 3-neuron Hopfield network of the form:

(4) $$\dot{x}=-x+W\varphi \left(x\right)$$

(5) $$\varphi \left(x_{i}\right)=\tanh\left(x_{i}\right)=\frac{e^{x_{i}}-e^{-x_{i}}}{e^{x_{i}}+e^{-x_{i}}}$$

where $x={\left[x_{1},x_{2},x_{3}\right]}^{T}$ is the neuron state vector, the neuron activation function $\varphi \left(x\right)={\left[\tanh\left(x_{1}\right),\tanh\left(x_{2}\right),\tanh\left(x_{3}\right)\right]}^{T}$, and synaptic weight matrix is:

(6) $$W=\left[\begin{array}{ccc} 2 & -1.58 & -0.27\\ 1.87 & 1.71 & 1.04\\ -6.92 & -0.58 & 1.1 \end{array}\right]$$

**Figure 6:**
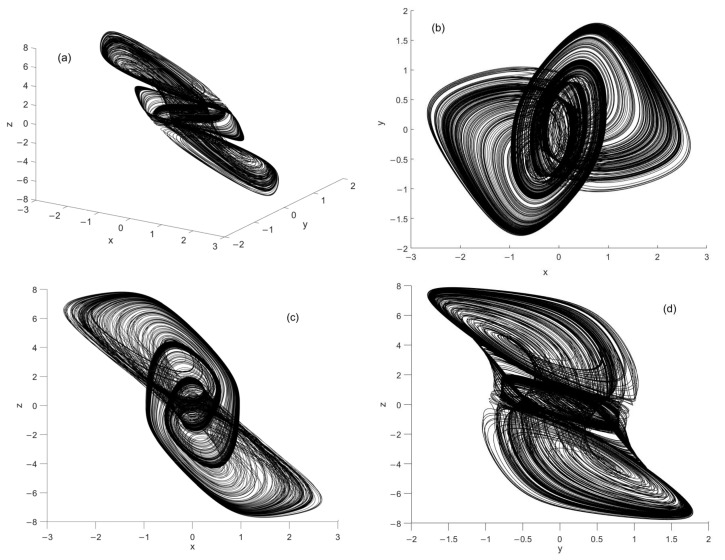
The double-scroll chaotic attractor of chaotic Hopfield neural network: (**a**) projection in x-y-z, (**b**) projection in x-y, (**c**) projection in x-z, (**d**) projection in y-z.

When the 3-neuron Hopfield network applies the weight matrix, the system can display chaotic behavior. The dynamic behavior of the chaotic Hopfield network is complex and suitable for image encryption. Figure 6 demonstrates the phrase portrait of the network with the initial state $\left[0.1,\;0.1,\;0.1\right]$, which shows a double-scroll chaotic attractor.

### 2.3. Public Key Cryptosystem

The RSA public key cryptosystem was proposed by Rivest et al. [45] in 1978, and its implementation depend on the difficulty of large integer decomposition. In RSA, users have their own public key $\left(N,e\right)$ and private key d. The key generation process is described as follows:

- Two large prime numbers (i.e., p and q) are generated randomly, and p≠q.
- The key N and Euler function $\varphi \left(N\right)$ are calculated as Equations (7) and (8):
  (7) N=p·q
  (8) $$\varphi \left(N\right)=\left(p-1\right)\cdot \left(q-1\right)$$
- An integer number e is generated as one of public keys obeyed Equations (9) and (10):
  (9) $$1<e<\varphi \left(N\right)$$
  (10) $$\gcd\left(e,\varphi \left(N\right)\right)=1$$
  where, gcd denotes the great common divisor.
- Then, d is calculated as Equation (11) as private key:
  (11) $$d=e^{-1}\;mod\;\varphi \left(N\right)$$
  where mod denotes the modulo operation.

After obtaining public key and private key, the plaintext is divided into multiple groups, each of which is a decimal number m of bit length less than N. The encryption operation can be described as Equation (12):

(12) $$C=m^{e}\;mod\;N$$

where C represents the ciphertext. The decryption operation is performed as Equation (13):

(13) $$m=C^{d}\;mod\;N$$

## 3. Algorithm Description

### 3.1. Encryption Steps

**Figure 7:**
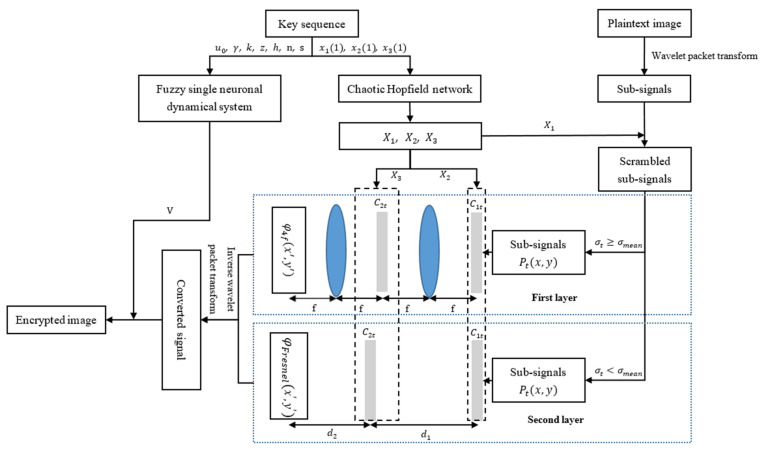
Diagram of the optical image encryption.

In this paper, an optical image encryption algorithm based on Hopfield neural network is proposed, as shown in Figure 7. To enhance the level of security, we use wavelet packet transform to decompose and filter the signal. Then, there are two layers in the subsequent encryption process. The DRPE method is applied to two layers through 4*f* system and Fresnel transform, respectively. Furthermore, RSA cryptosystem is performed for key-sequence management. It should be noted that there is no specific method and limitation for random matrix construction in the traditional DRPE. Thus, we construct random-phase masks to encrypt the decomposed signal by different chaotic sequences. The detail of the process is described in Figure 7.

Suppose the size of plaintext image is M×N, where M is the length of the row and N is the length of the column.

Step 1: The plaintext image is decomposed using m order wavelet packet transform, and sub-signals are obtained. Each sub-signal has a corresponding number, ranging from 1 to T. Set $x_{1}\left(1\right)$, $x_{2}\left(1\right)$, $x_{3}\left(1\right)$ as the initial values of the Hopfield chaotic neural network, and the M×N times iteration is performed to get three sequences.

Step 2: Calculate the state variable s by Equation (14). When S=0, insert $\left\{x_{1},x_{2},x_{3}\right\}$ to new sequences. When S=1, insert $\left\{x_{2},x_{3},x_{1}\right\}$ to new sequences. When S=2, insert $\left\{x_{3},x_{1},x_{2}\right\}$ to new sequences. After M×N iterations, three new sequences $X_{1}$, $X_{2}$, $X_{3}$ are obtained.

(14) $$S=mod(floor(\sqrt{x_{1}^{2}+x_{2}^{2}+x_{3}^{2}},3))$$

**Figure 8:**
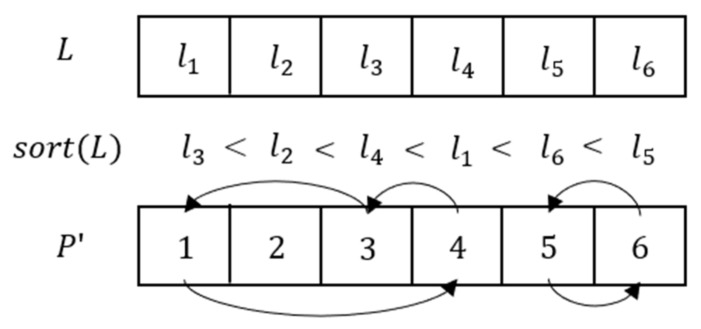
Scrambling process of matrix.

Step 3: The sequence $X_{1}$ is divided into subsequences [L1, L2, …, LT], and each sub-signal is converted into a 1D matrix [P1, P2, …, PT]. Sort each sequence L in ascending order, and matrix $P^{\prime }$ is obtained according to the sorting result. The process is shown in Figure 8. Then, $P^{\prime }$ is converted back to a 2D matrix.

Step 4: The standard deviation $\sigma_{t}$ of each scrambled sub-signal is calculated, and the mean of [$\sigma_{1},\ldots ,\sigma_{t},\ldots ,\sigma_{T}$] is obtained. If $\sigma_{t}\geq \sigma_{mean}$, the sub-signal is assigned to the first layer. If $\sigma_{t}<\sigma_{mean}$, the sub-signal is assigned to the second layer.

Step 5: The sequence $X_{2}$ and $X_{3}$ is divided into subsequences numbered from 1 to T, respectively. Each subsequence is converted into matrix with the size of sub-signal. Then, perform Arnold scrambling on each chaotic matrix as in Equation (15):

(15) $$\left[\begin{array}{c} x^{\prime }\\ y^{\prime } \end{array}\right]=\left[\begin{array}{cc} 1 & \alpha \\ \beta & \alpha \beta +1 \end{array}\right]\left[\begin{array}{c} x\\ y \end{array}\right]\;mod\;N$$

where $\left(x,y\right)$ is the original coordinate, $\left(x^{\prime },y^{\prime }\right)$ is the scrambled coordinate.

Step 6: After Arnold scrambling, normalize each matrix from $X_{2t}$ to obtain chaotic random matrix $g_{t}\left(x,y\right)$, and normalize each matrix from $X_{3t}$ to obtain chaotic random matrix $r_{t}\left(x,y\right)$. Then construct chaotic random phase:

(16) $$C_{1t}\left(x,y\right)=\exp\left[i2\pi g_{t}\left(x,y\right)\right]$$

(17) $$C_{2t}\left(x,y\right)=\exp\left[i2\pi r_{t}\left(x,y\right)\right]$$

Step 7: Perform DRPE of 4*f* system on sub-signals of the first layer as in Equation (18). Then, DRPE of Fresnel transform is performed on sub signals of the second layer as in Equation (19):

(18) $$\varphi_{4f}\left(x^{\prime },y^{\prime }\right)=FT^{-1}\left\{FT\left[P_{t}\left(x,y\right)\cdot C_{1t}\left(x,y\right)\right]\cdot C_{2t}\left(x,y\right)\right\}$$

(19) $$\varphi_{Fresnel}\left(x^{\prime },y^{\prime }\right)=FrT_{\rho ,d1}\left\{FrT_{\rho ,d2}\left[P_{t}\left(x,y\right)\cdot C_{1t}\left(x,y\right)\right]\cdot C_{2t}\left(x,y\right)\right\}$$

where $\left(x,y\right)$ is the original coordinate of sub-signal, $\left(x^{\prime },y^{\prime }\right)$ is the coordinate after DRPE in 4*f* system or Fresnel transform, ρ and is the incident light wavelength, $d_{1}$ and $d_{2}$ represent the diffraction distance. $FT\left[\cdot \right]$ and $FT^{-1}\left[\cdot \right]$ represent Fourier transform and inverse Fourier transform, respectively. $FrT\left[\cdot \right]$ represents Fresnel transform.

Step 8: Sub-signals are transformed into M×N matrix E by m order inverse wavelet packet transform. Then, the complex-value matrix E is normalized.

Step 9$:\;u_{0}$, γ, k, z, h, n and s are initial value and system parameters of fuzzy single neuronal dynamic system, therefore they are used as key sequence. Iterate fuzzy single neuronal dynamic system M×N times, and a chaotic sequence V is obtained. This sequence is used to scramble matrix E to obtain an encrypted image; the process is the same as Step 3.

The keys used in the process of encryption are divided into three sequences. The first sequence includes initial values of chaotic Hopfield neural network and parameters of Arnold scrambling (i.e., $x_{1}\left(1\right)$, $x_{2}\left(1\right)$, $x_{3}\left(1\right)$, α, β). The second sequence consists of initial value and system parameters of fuzzy single neuronal dynamic system (i.e., $u_{0}$, γ, k, z, h, n, s). The third sequence is composed of wavelet packet transform order, incident light wavelength and diffraction distance (i.e., m, ρ, $d_{1}$, $d_{2}$). The ciphertext sequences are obtained by RSA cryptosystem using public keys N and e.

### 3.2. Image Decryption

In this work, keys used in the scheme are integrated and hidden by an RSA cryptosystem. Thus, the process of decryption can be performed for cases where three key sequences are retrieved. The users can restore the sequences to perform the decryption process according to private keys N and d.

## 4. Experimental Results and Security Analysis

### 4.1. Experimental Results

**Figure 9:**
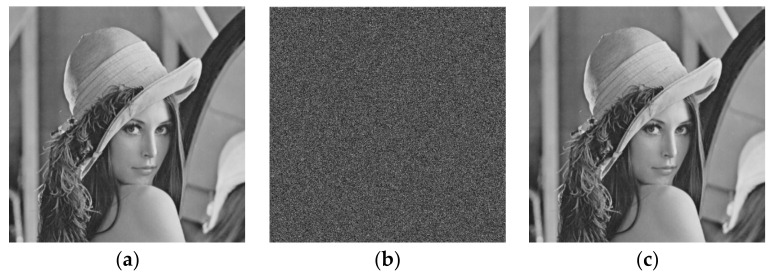
Encryption experiment results: (**a**) original image; (**b**) encrypted image; (**c**) decrypted image.

The numerical simulation and security verification of the algorithm are performed by Matlab R2017a. A standard grayscale image Lena of size 512×512 is shown in Figure 9a is the original image. The initial values and system parameters of the algorithm are m=2, $x_{1}\left(1\right)=0.1$, $x_{2}\left(1\right)=0.1$, $x_{3}\left(1\right)=0.1$, $u_{0}=0.1$, γ=−250, k=−0.6, z=−0.1, h=0.01, n=14, s=0.1, ρ=632.8 nm, $d_{1}=40\;\mathrm{mm}$, $d_{2}=50\;\mathrm{mm}$, α=3, β=5, respectively. In addition, two prime numbers (p=257 and q=311) are applied in the RSA cryptosystem to obtain public key (N=79,927, e=6937) and private key (d=4393). Figure 9b shows the encrypted grayscale image Lena, and the decrypted image with correct keys is shown as Figure 9c.

### 4.2. Security Analysis

#### 4.2.1. Key Space Analysis

In this work, the precision of noninteger key is considered as $10^{-16}$. This algorithm covers a chaotic Hopfield neural network with 3 noninteger initial values, fuzzy single neuronal dynamic system with 7 noninteger initial values and other system parameters. Thus, the key space is larger than $2^{128}$, which is enough to resist brute force attacks [46,47].

#### 4.2.2. Sensitivity Analysis

**Figure 10:**
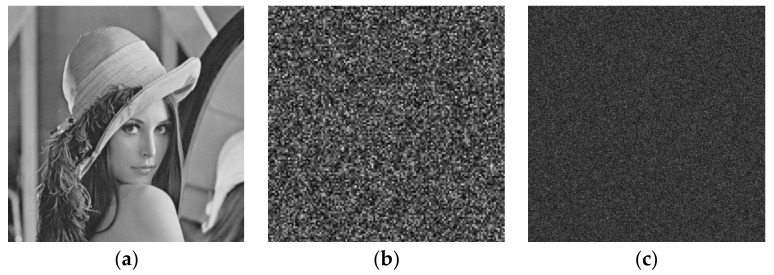
Encryption experiment results: (**a**) original image; (**b**) decrypted image with initial deviation $10^{-16}$ of $x_{1}\left(1\right)$; (**c**) decrypted image with initial deviation $10^{-16}$ of $u_{0}$.

In order to test key sensitivity, the influence of varying initial values and system parameters on the decryption result is explored. When the initial deviation of the chaotic Hopfield neural network or fuzzy single neuronal dynamic system is $10^{-16}$, the generated sequence and random phase masks cannot correctly decrypt the image, as shown in Figure 10.

In addition, the correlation coefficient (CC) is used as the criterion for quantitative analysis of the difference between the original image and decrypted image.

(20) $$\mathrm{CC}=\frac{\left|\sum_{i=1}^{m}\sum_{j=1}^{n}\left[f_{1}\left(i,j\right)-E\left(f_{1}\left(i,j\right)\right)\right]\left[f_{2}\left(i,j\right)-E\left(f_{2}\left(i,j\right)\right)\right]\right|}{\sqrt{\sum_{i=1}^{m}\sum_{j=1}^{n}{\left[f_{1}\left(i,j\right)-E\left(f_{1}\left(i,j\right)\right)\right]}^{2}}\sqrt{\sum_{i=1}^{m}\sum_{j=1}^{n}{\left[f_{2}\left(i,j\right)-E\left(f_{2}\left(i,j\right)\right)\right]}^{2}}}$$

where $f_{1}\left(i,j\right)$ represents the plaintext image, $f_{2}\left(i,j\right)$ represents the recovered image, and $E\left(\cdot \right)$ represents the expected value operation.

**Figure 11:**
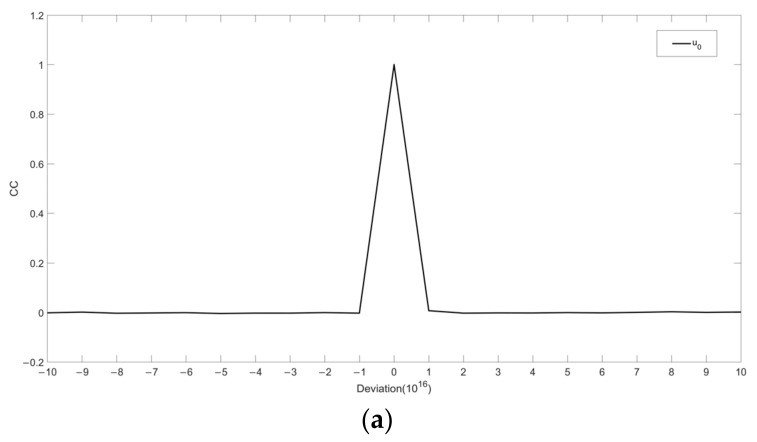

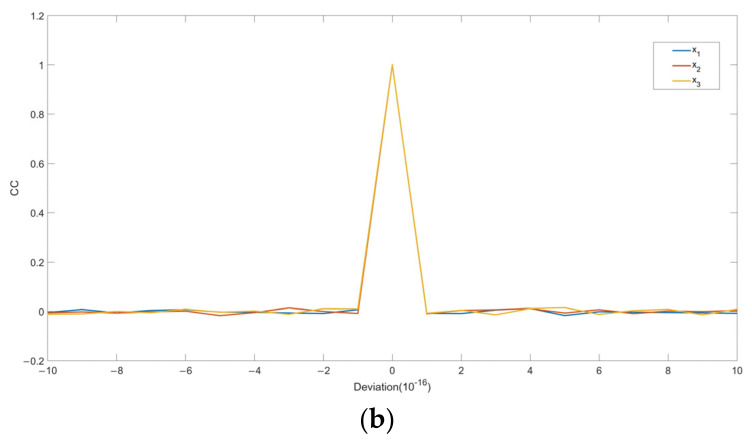
Sensitivity analysis of the keys of chaotic systems: (**a**) decrypted CC curve of the key $u_{0}$; (**b**) decrypted CC curve of the keys $x_{1}\left(1\right)$, $x_{2}\left(1\right)$ and $x_{3}\left(1\right)$.

The relationship between CC of the decrypted image and initial values is obtained, as shown in Figure 11. It can be seen that any information about the plaintext image cannot be retrieved when keys change slightly. Thus, the sensitivity of the algorithm is qualified.

#### 4.2.3. Correlation Analysis

Due to the discernibility of information in plaintext images, adjacent pixels are usually highly correlated. Therefore, the reduction of the correlation between adjacent pixels of the cipher images is necessary [48]. The calculation of pixel correlation is shown as Equation (21).

(21) $$\left\{\begin{array}{c} \bar{x}=\frac{1}{N}\overset{N}{\underset{i=1}{\sum }}x_{i}\\ D\left(x\right)=\frac{1}{N}\overset{N}{\underset{i=1}{\sum }}x_{i}-\bar{x}\\ cov\left(x,y\right)=\frac{1}{N}\overset{N}{\underset{i=1}{\sum }}\left(x_{i}-\bar{x}\;\right)\left(y_{i}-\bar{y}\;\right)\\ \rho_{xy}=\frac{cov\left(x,y\right)}{\sqrt{D\left(x\right)}\sqrt{D\left(y\right)}} \end{array}\right.$$

where $x_{i}$ and $y_{i}$ represent the values of adjacent pixels, and $\rho_{xy}$ denotes the correlation between adjacent pixels. The results of correlation coefficients of plaintext images and cipher images in horizontal direction, vertical direction and diagonal direction are shown in Table 1.

It should be noted that the scheme combines double random phase encoding in 4*f* system and Fresnel domain with Hopfield neural network to address inherent limitation of random matrix construction. The correlation coefficients of four encrypted images using various schemes are also demonstrated in Table 1. It can be seen that our method reaches relatively low correlation coefficients compared with other methods, which indicates that the integration of double random phase encoding and Hopfield neural network can achieve better performance.

#### 4.2.4. Histogram Analysis

**Figure 12:**
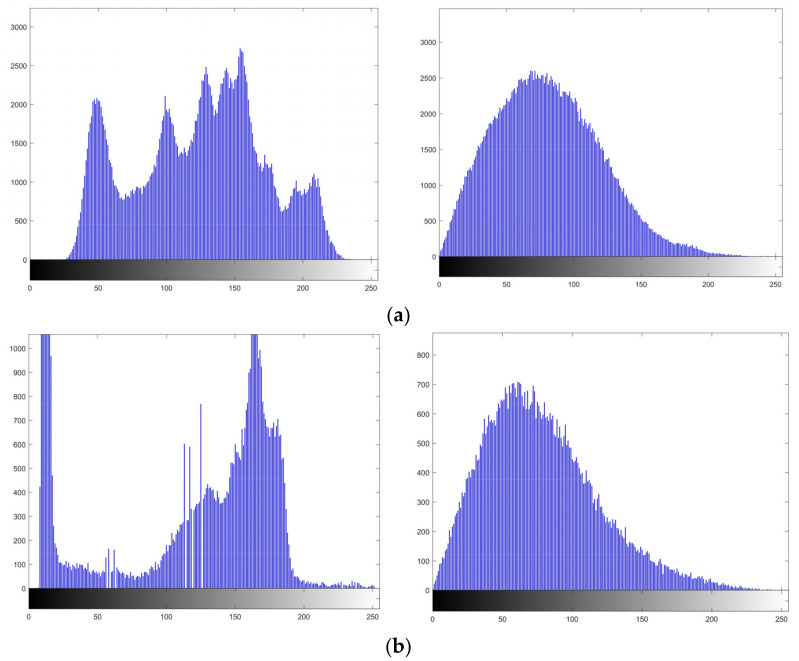

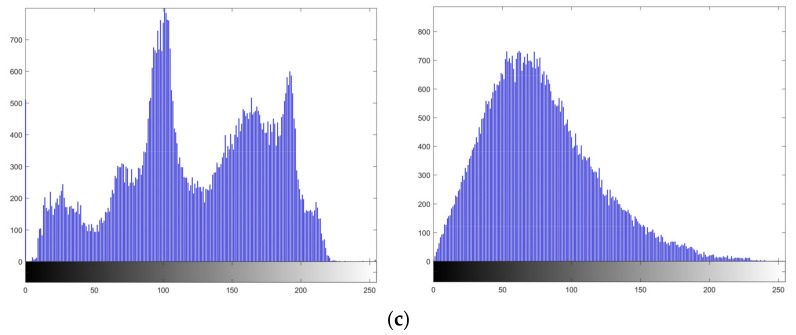
Histogram analysis of the plain images and cipher images: (**a**) Lena; (**b**) Cameraman; (**c**) Peppers.

Histogram analysis is the statistic of the number of times each value appears, in order to demonstrate the distribution of pixel values [26]. The histogram of cipher image should not reflect any information about the original image. Figure 12 shows the histogram analysis of four images. It can be seen that the histograms of encrypted test images approximate Rayleigh distribution function, therefore the frequency distribution of plaintext images is hidden.

#### 4.2.5. Binary Image Test

**Figure 13:**
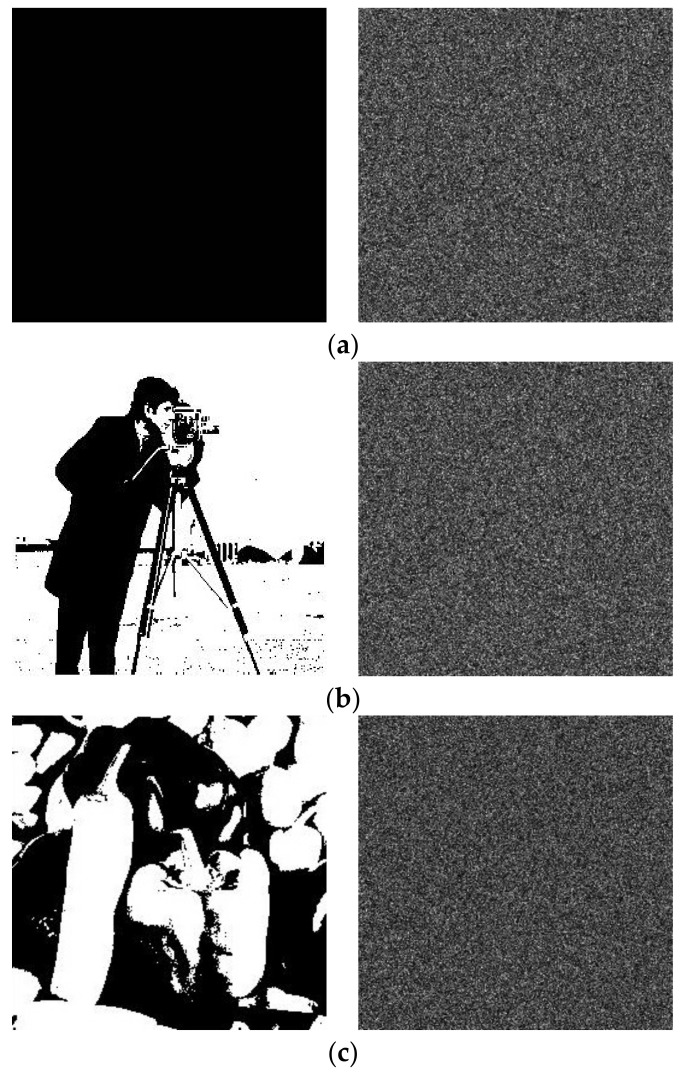
Binary images test: (**a**) black image and corresponding encrypted image; (**b**) cameraman and corresponding encrypted image; (**c**) peppers and corresponding encrypted image.

Due to the simple content of binary images, the traditional methods are not applicable sometimes. To test the performance of the algorithm on binary images, the results of encryption are shown in Figure 13. It can be seen that our algorithm works well on binary images, and the correlation coefficients of cipher images are listed in Table 2.

#### 4.2.6. Noise Attack

**Figure 14:**
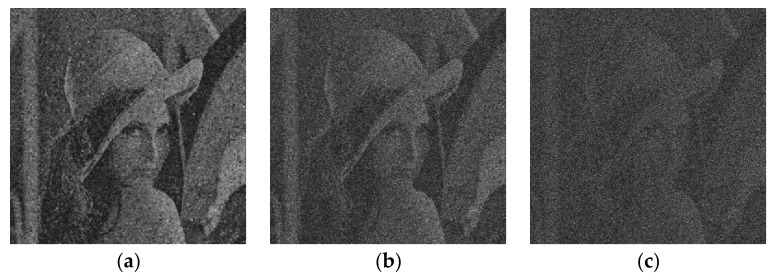
Decrypted Lena obtained with (**a**) k=0.01; (**b**) k=0.05; (**c**) k=0.1.

**Figure 15:**
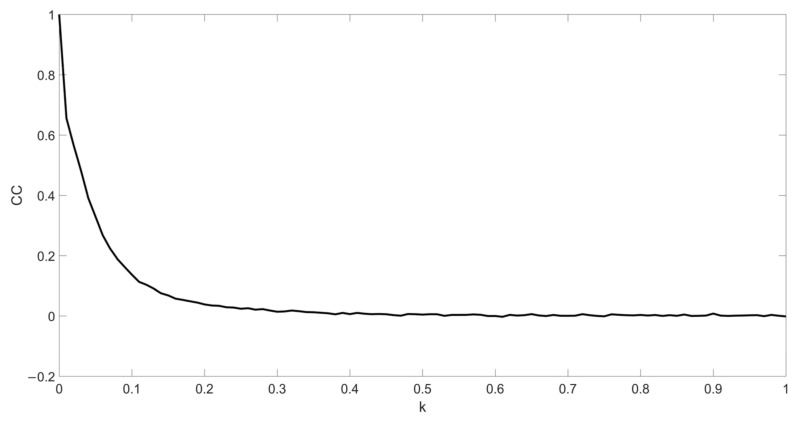
The CC curve of noise attack.

In practice, the cipher images may be affected by noise. We consider the robustness of our algorithm against noise by polluting the encrypted images of Lena with Gaussian random noise, which is expressed as:

(22) $$M^{\prime }\left(x,y\right)=M\left(x,y\right)\times \left(1+kG\left(x,y\right)\right)$$

where $M\left(x,y\right)$ denotes the original cipher image, $M^{\prime }\left(x,y\right)$ denotes the noise-affected cipher image, *k* is the noise strength and $G\left(x,y\right)$ is the Gaussian random noise with zero-mean and variance 1. The decrypted images with the noise intensity *k* = 0.01, *k* = 0.05 and *k* = 0.1 are shown in Figure 14. The CC value changing with the noise strength is shown in Figure 15. It can be seen that the contour of original image can be distinguished from the decrypted image.

#### 4.2.7. Comparative Analysis

The comparative analysis among different schemes is demonstrated in Table 3. The experimental environments are as follows: Matlab R2017a, AMD Ryzen 5 3600 6-Core Processor 3.60 GHz with 16 GB memory and Windows 10 Operation System, and grayscale Pepper is used as the plaintext image. Table 3 shows that our method reaches the lowest running time in the decryption process, and the running time is slightly higher than [50] in the encryption process. In addition, CC values between the original image and decrypted image are listed in Table 3. It can be seen that the scheme in this study achieves the highest CC value, which is related to the more accurate reconstruction of random phase mask by using the chaotic Hopfield neural network.

## 5. Conclusions

This paper proposes an optical image encryption method using double random-phase encoding in 4*f* system and Fresnel domain based on chaotic system. The chaotic sequences are constructed by applying a fuzzy single neuronal dynamic system and a Hopfield chaotic neural network. The plaintext image is decomposed into sub-signals by wavelet packet transform, and then the sub-signals are scrambled. By adaptive classification, the sub-signals are divided into two layers. The first layer and second layer are encrypted in 4*f* system and Fresnel domain, thus completing the hybrid encryption. After inverse wavelet packet transform, the encrypted image is obtained through another scrambling. The RSA cryptosystem is applied to the allocation and management of the keys used in the scheme. Numerical simulations have demonstrated the security and effectiveness of the proposed scheme. The suggested scheme implements selective encryption of the local image, which improves the protection efficiency of vital information. It should be noted that the fuzzy numbers effectively enhance the stability and key space of the single neuronal dynamic system, and different fuzzy numbers other than triangular may be applicable to more chaotic systems. In addition, the feasibility of combining the chaotic Hopfield neural network with optical methods to construct an image encryption scheme has been verified. In further work, the application of other neural networks or chaotic systems may have more positive effects on optical image encryption.

## Figures and Tables

**Table 1:** Correlation coefficients of plain images and cipher images in horizontal direction, vertical direction and diagonal direction.

Images		Correlation Coefficient		
		Horizontal	Vertical	Diagonal
Lena (512×512)	Plain image	0.9850	0.9719	0.9593
	Cipher image (our scheme)	−0.0005	−0.0033	−0.0009
	Cipher image [49]	0.9407	−0.0273	−0.0140
	Cipher image [50]	−0.0097	0.0032	−0.0051
	Cipher image [51]	−0.0084	−0.0017	−0.0019
	Cipher image [52]	−0.0023	0.0028	−0.0030
Cameraman (256×256)	Plain image	0.9592	0.9340	0.9089
	Cipher image (our scheme)	−0.0004	−0.0003	0.0030
	Cipher image [49]	0.9176	−0.0175	−0.0312
	Cipher image [50]	−0.0186	0.0053	0.0095
	Cipher image [51]	0.0208	0.0009	0.0021
	Cipher image [52]	0.0005	−0.0034	0.0008
Peppers (256×256)	Plain image	0.9651	0.9759	0.9457
	Cipher image (our scheme)	−0.0007	−0.0009	0.0041
	Cipher image [49]	0.9235	−0.0304	−0.0240
	Cipher image [50]	−0.0247	−0.0129	−0.0031
	Cipher image [51]	−0.0131	0.0024	0.0002
	Cipher image [52]	−0.0027	0.0010	−0.0069
Baboon (256×256)	Plain image	0.8003	0.8763	0.7627
	Cipher image (our scheme)	0.0015	−0.0030	0.0007
	Cipher image [49]	0.9323	−0.0482	−0.0306
	Cipher image [50]	−0.0155	−0.0251	0.0013
	Cipher image [51]	0.0026	−0.0015	0.0014
	Cipher image [52]	−0.0060	−0.0064	−0.0050

**Table 2:** Correlation coefficient of encrypted binary images.

Images		Correlation Coefficient		
		Horizontal	Vertical	Diagonal
Black (256×256)	Cipher image	0.0035	−0.0017	0.0022
Cameraman (256×256)	Cipher image	−0.0033	0.0007	0.0040
Peppers (256×256)	Cipher image	−0.0050	0.0010	−0.0030

**Table 3:** Comparative analysis of different schemes.

Scheme	Encryption Time (s)	Decryption Time (s)	CC
[50]	0.1136	0.2031	0.9998
[51]	0.4111	0.4259	0.9996
[53]	0.2974	0.3708	0.9832
Our scheme	0.1249	0.1596	0.9999

## Data Availability

Not applicable.
